# Effect of multiple set on intramuscular metabolic stress during low-intensity resistance exercise with blood flow restriction

**DOI:** 10.1007/s00421-012-2377-x

**Published:** 2012-03-14

**Authors:** Tadashi Suga, Koichi Okita, Shingo Takada, Masashi Omokawa, Tomoyasu Kadoguchi, Takashi Yokota, Kagami Hirabayashi, Masashige Takahashi, Noriteru Morita, Masahiro Horiuchi, Shintaro Kinugawa, Hiroyuki Tsutsui

**Affiliations:** 1 Department of Cardiovascular Medicine, Hokkaido University Graduate School of Medicine, Sapporo, Japan; 2 The Japan Society for the Promotion of Science, Tokyo, Japan; 3 Graduate School of Program in Lifelong Learning Studies, Hokusho University, 23 Bunkyodai, Ebetsu, Hokkaido 069-8511 Japan; 4 Northern Regions Lifelong Sports Research Center (SPOR), Ebetsu, Japan; 5 Department of Sports Education, Hokkaido University of Education, Iwamizawa, Japan

**Keywords:** Energetic metabolism, Resistance training, Magnetic resonance spectroscopy, Muscle hypertrophy

## Abstract

Our previous study reported that intramuscular metabolic stress during low-intensity resistance exercise was significantly enhanced by combining blood flow restriction (BFR); however, they did not reach the levels achieved during high-intensity resistance exercise. That study was performed using a single set of exercise; however, usual resistance exercise consists of multiple sets with rest intervals. Therefore, we investigated the intramuscular metabolic stress during multiple-set BFR exercises, and compared the results with those during multiple-set high-intensity resistance exercise. Twelve healthy young subjects performed 3 sets of 1-min unilateral plantar flexion (30 repetitions) with 1-min intervals under 4 different conditions: low intensity (L, 20 % 1 RM) and high intensity (H, 65 % 1 RM) without BFR, and L with intermittent BFR (IBFR, only during exercise) and with continuous BFR (CBFR, during rest intervals as well as exercise). Intramuscular metabolic stress, defined as intramuscular metabolites and pH, and muscle fiber recruitment were evaluated by ^31^P-magnetic resonance spectroscopy. The changes of intramuscular metabolites and pH during IBFR were significantly greater than those in L but significantly lower than those in H. By contrast, those changes in CBFR were similar to those in H. Moreover, the fast-twitch fiber recruitment, evaluating by a splitting Pi peak, showed a similar level to H. In conclusion, the multiple sets of low-intensity resistance exercise with continuous BFR could achieve with the same metabolic stress as multiple sets of high-intensity resistance exercise.

## Introduction

Resistance training with high-intensity mechanical load can achieve muscle hypertrophy and strength increase; however, it generates intensive stress in musculo-skeletal and cardiovascular systems. Such high-intensity resistance training may induce orthopedic and cardiovascular problems. Thus, adequate high-intensity loads cannot be applied to elderly people and patients with various disorders (Williams et al. 2007). Despite using low-intensity mechanical loads, resistance training with blood flow restriction (BFR) has resulted in beneficial training effects (Clark et al. 2011; Karabulut et al. 2010; Takarada et al. 2002, 2004), which may be appropriate for physical fitness and rehabilitation in older people and those recovering from illness (Karabulut et al. 2010, 2011; Ohta et al. 2003; Ozaki et al. 2011). In previous studies, it has speculated that low-intensity resistance exercise with BFR might provide enhanced intramuscular metabolic stress and added skeletal muscle fiber recruitment (Fujita et al. 2007; Takarada et al. 2002, 2004); however, the exact mechanisms were not fully clarified.

In our previous studies (Suga et al. 2009, 2010), metabolic stress and muscle fiber recruitment during low-intensity resistance exercise were significantly increased with the addition of BFR; however, the effects of this combination did not reach the level of those during high-intensity resistance exercise. The previous studies (Suga et al. 2009, 2010) used a single set of exercise; however, the usual resistance training involves multiple sets with rest intervals (Kraemer and Ratamess 2004). In fact, multiple-set resistance training generally results in greater training effects than single-set (Krieger 2009, 2010).

The application of BFR during rest intervals in an exercise session is an important training variable, along with exercise intensity and BFR pressure (Cook et al. 2007). Continuous BFR during rest interval as well as exercise in multiple-set BFR exercise may be key factors for increasing metabolic stress and muscle fiber recruitment in skeletal muscle. Therefore, we evaluated the metabolic stress and fast-twitch (FT) fiber recruitment during multiple-set low-intensity BFR exercise, and then examined the effects of the BFR mode, either intermittent or continuous. Moreover, those BFR exercise protocols were compared to multiple-set high-intensity resistance exercise.

## Methods

### Subjects

Twelve younger male subjects (age 22 ± 4 years, height 172 ± 7 cm, weight 69 ± 7 kg, mean ± SD) participated in the study. All subjects were healthy and without orthopedic or cardiovascular diseases. Subjects were tested on two occasions, separated by at least 48 h. The subjects were instructed to refrain from transient strenuous physical activity and alcohol for at least 24 h prior to the testing. On the experiment day, subjects were required to abstain from caffeine and to fast for 4 h before the trial. The experimental trails were started from ~1,800 h and then were arranged to be performed at the same time for each subject on the second experimental day. Informed consent was obtained from all subjects. This study was approved by the Ethics Committee of Hokusho University.

### Exercise protocols

Subjects randomly performed the right ankle plantar flexion exercises with four different exercise conditions in two experimental days. Each experimental exercise session consisted of 3 sets of 1 min-exercise with 30 repetitions per set, lifting the weight 5 cm above ground. The rest intervals between sets were 1 min (Goto et al. 2005; Kraemer et al. 1990; Takarada et al. 2004). Thus, each experimental exercise session lasted 5 min. Four exercise conditions were set as follows: two resistance exercises without BFR and two BFR exercise protocols. The two resistance exercises without BFR were low-intensity (L) and high-intensity (H) exercises, at 20 and 65 % 1 RM, respectively (Suga et al. 2009, 2010). The two BFR protocols were performed with intermittent or continuous BFR combined with L. In the intermittent BFR (IBFR) protocol, the BFR was applied only during exercise. The pressure cuff for IBFR was inflated before every set and released after the finish of sets. In the continuous BFR (CBFR) protocol, the BFR was maintained throughout the 5-min exercise session. The four exercise conditions were randomly performed on two exercise conditions per day. On each experimental day, the interval between conditions was at least 30 min. Moreover, before the implementation of the second condition, the recovery in the subject’s altered intramuscular metabolites and pH to baseline levels was confirmed. The subject’s 1 RM was determined as successful contraction on the same plantar flexion apparatus equipped with a magnetic resonance device in the first experimental day. The 1 RM trials were designed using increments of 10 kg until 60–80 % of the perceived maximum. Then, the load was gradually increased by 1–5 kg weights until lift fails, in which the subject was not able to maintain proper form, or to completely lift the weight. The last acceptable lift with the highest possible load was determined as 1 RM. Mean ± SD of resistance loads at 20 and 65 % 1 RM were 10 ± 1 and 33 ± 4 kg, respectively. BFR was carried out using a pneumatic rapid inflator (E-20 rapid cuff inflator, Hokanson, USA) with an 18.5-cm-wide pressure cuff placed around the right thigh. BFR pressure was applied 130 % of the subject’s resting systolic blood pressure (Clark et al. 2011; Cook et al. 2007; Takano et al. 2005; Suga et al. 2009, 2010) and 144 ± 21 mmHg on average.

### ^31^P-magnetic resonance spectroscopy

Subjects lay in the supine position on an original apparatus equipped with a magnetic resonance device, and the right foot was coupled to the pedal by a Velcro strap. ^31^P-MRS was performed using a 55-cm bore, 1.5-tesla superconducting magnet (Magnetom Vision VB33G, Siemens Erlangen, Germany). An 80-mm surface coil was placed under the muscle belly of the right gastrocnemius. Shimming was adjusted using the proton signal from water. Spectra of high-energy phosphate were acquired at a pulse width of 500 μs, a transmitter voltage of 20 V and a repetition time of 2,000 ms. The spectra were obtained at rest and during exercise. Each spectrum consisted of an average of 8 scans during 16 s before each time point. Peaks corresponding to high-energy phosphates were determined based on the chemical shifts. Peak areas were automatically calculated by peak fitting and integration after baseline correction using MR software. The phosphocreatine (PCr) millimolar concentration ([PCr]) assumed that [PCr] + creatine concentration ([Cr]) = 42.5 mM (Harris et al. 1974) and supposed that inorganic phosphate (P_i_) concentration ([P_i_]) is equal to [Cr] (Kemp and Radda 1994). The total change in [PCr] through the exercise sessions was quantified from the integrated area of the [PCr]–time curve. Diprotonated phosphate (H_2_PO_4_^−^) was calculated using [P_i_] (Lanza et al. 2005, 2007). Intramuscular pH was calculated from the chemical shift of Pi relative to PCr. When distinct Pi splitting was shown, the pH was calculated by standardizing the obtained individual pH on the basis of peaks corresponding to each Pi (Lanza et al. 2005, 2007).

### Statistical analyses

Two-way ANOVA with repeated measures was used for comparisons among exercise conditions. Post hoc comparisons were made by Bonferroni’s test. The comparisons of split-peak Pi appearance among exercise conditions were performed by a χ^2^ test. The level of significance was set at *P* < 0.05. All statistical tests were performed using SPSS 13.0 for Windows software.

## Results

**Fig. 1:**
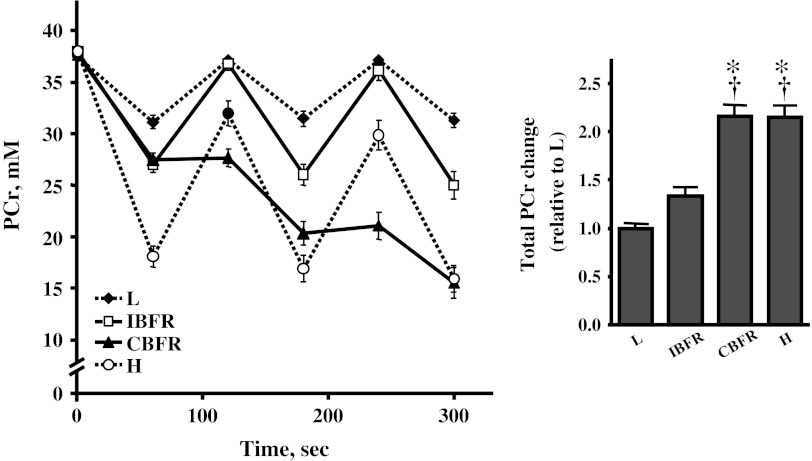
Time course of phosphocreatine (PCr) concentration during exercise protocols *Symbols* indicate means and *error bars* indicate SE. Significant difference between condition;*P < 0.05 versus *L*, ^†^ *P* < 0.05 versus *IBFR*

At the end of the final sets in all four exercise conditions, intramuscular metabolites were significantly changed compared with resting values (respectively, *P* < 0.001). Intramuscular pH at the end of the final set showed a significant decrease in two BFR protocols and H (*P* < 0.001), but not in L, in comparison with that at rest. Changes of intramuscular metabolites and pH at the end of the final set in IBFR were significantly greater than those in L but significantly less than those in H (Table 1; Fig. 1). Those changes in CBFR were significantly greater than in IBFR (*P* < 0.001), and similar to H (Table 1; Fig. 1). The total change (the integrated area of the [PCr]–time curve) in PCr through each 5-min exercise session tended to be higher in IBFR than in L (*P* = 0.074), but the difference did not reach statistical significance (Fig. 1). Total PCr change in CBFR was significantly greater than in IBFR (*P* < 0.001) and similar to H (Fig. 1).

**Table 1:** Intramuscular metabolite concentrations and pH at rest and the end of final set

	L	BFR protocols		H
		IBFR	CBFR	
Pi, mM				
Rest	5.1 ± 0.2	4.6 ± 0.2	4.8 ± 0.3	4.5 ± 0.3
End of final set	11.2 ± 0.7*	17.5 ± 1.3*^†^	27.0 ± 1.5*^†‡^	26.6 ± 1.3*^†‡^
H_2_PO_4_^−^, mM				
Rest	1.8 ± 0.3	1.6 ± 0.3	1.7 ± 0.4	1.6 ± 0.3
End of final set	3.9 ± 0.9*	6.8 ± 2.6*^†^	12.2 ± 3.0*^†‡^	11.6 ± 2.9*^†‡^
Intramuscular pH				
Rest	7.01 ± 0.01	7.01 ± 0.02	7.01 ± 0.02	7.00 ± 0.02
End of final set	7.03 ± 0.02	6.96 ± 0.07*^†^	6.84 ± 0.07*^†‡^	6.87 ± 0.07*^†‡^

Values are mean ± SD

*BFR* blood flow restriction, *L* low-intensity resistance exercise at 20 % 1RM, *H* high-intensity resistance exercise at 65 % 1RM, *IBFR* intermittent BFR exercise protocol, *CBFR* continuous BFR exercise protocol

* Significant difference (*P* < 0.05) between rest and the end of final, ^†^significant difference (*P* < 0.05) from *L*, ^‡^significant difference (*P* < 0.05) from IBFR

**Fig. 2:**
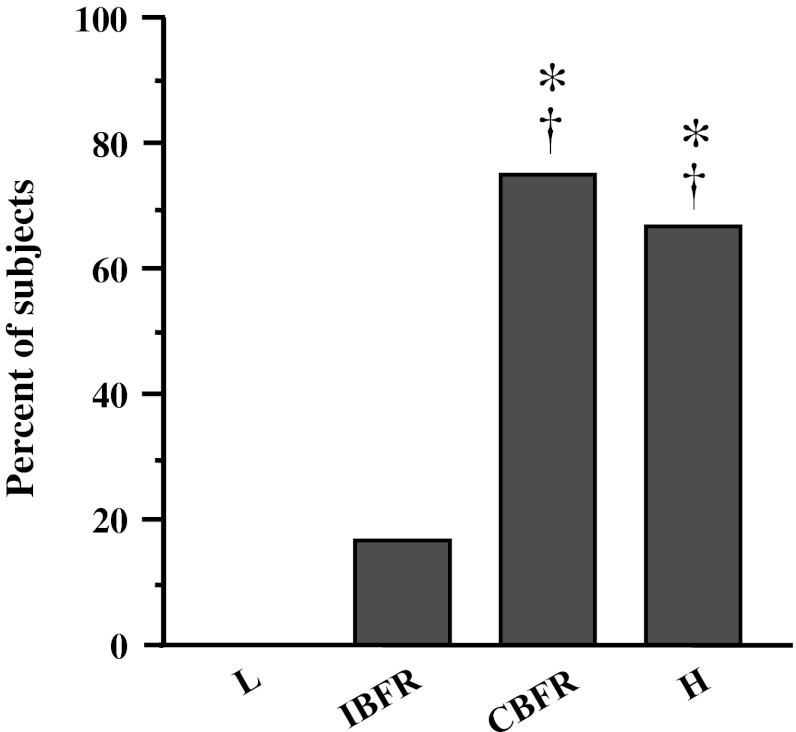
The number of subjects who showed split-peak Pi. *P < 0.05 versus *L*, ^†^ *P* < 0.05 versus *IBFR*

The splitting of Pi peaks, representing the recruitment of FT fiber (Park et al. 1987; Vandenborne et al. 1991), was observed in two BFR protocols and H, but not in L. The number of subjects who showed split Pi peak during the final set in CBFR was significantly higher than that in L and IBFR (*P* < 0.001) and similar to that in H (Fig. 2).

## Discussion

The present study investigated the effect of multiple-set BFR exercise on intramuscular metabolic stress and skeletal muscle fiber recruitment, and then examined the effects of the different BFR mode, intermittent and continuous. The results showed that metabolic stress, namely intramuscular metabolites and pH, was significantly increased using intermittent restriction in the BFR protocol, but did not reach that of high-intensity resistance exercise. This result agrees with the result from a single set in our previous studies (Suga et al. 2009, 2010). However, the use of continuous restriction throughout the exercise session in the BFR protocol resulted in metabolic stress equivalent to that of high-intensity resistance exercise. The enhancement in FT fiber recruitment indicated as split Pi peak during exercise showed the same result. Thus, it was suggested that the multiple-set BFR exercise with continuous BFR might increase intramuscular metabolic stress and enhance skeletal muscle fiber recruitment equally to multiple-set high-intensity resistance exercise.

Harris and coworkers (Harris et al. 1976) found that the changes of intramuscular metabolites and pH after exercise are maintained by applying ischemic cuff pressure; this is called a “metabolic freeze” (Okita et al. 1998). Metabolic freeze method might be a useful tool to enhance training effect in BFR training. In the result of our present study, the changes in intramuscular metabolites and pH induced by exercise could be retained by continuing BFR during rest intervals. Such a technique might play an important role in promoting metabolic stress during BFR exercise. Accumulating evidence suggests that high-metabolic accumulation during resistance exercise can strongly stimulate growth hormone (GH) release and skeletal muscle growth (Goto et al. 2005; Kraemer et al. 1990; Takarada et al. 2002). For example, Kraemer et al. (1990) and other studies (Takarada et al. 2002) have shown that a resistance exercise protocol with short rest intervals could effectively enhance the post-GH response and muscular training adaptations. This method of resistance training has something in common with the continuous BFR protocol in terms of inhibiting metabolic recovery during rest intervals. Thus, we propose that the manipulation of rest intervals in resistance exercise might play a key role for obtaining successful training effects.

In our previous study (Suga et al. 2009), splitting of Pi peaks during a single set of BFR protocol using the same intensity and pressure as that in the present study was observed in only 31 % of subjects. This percent was significantly lower than that (70 %) during high-intensity resistance exercise. By contrast, the present findings showed that the number of subjects showing split-peak Pi during multi-set BFR protocol with continuous BFR was progressively increased as the number of sets, reaching a rate of 75 % of subjects in the final set of exercise, despite the use of the same typical exercise intensity and BFR pressure as in our previous study (Suga et al. 2009). Moreover, this incidence was the same as that in multiple-set high-intensity resistance exercise. Thus, by applying continuous restriction, multiple-set BFR exercise not only increases metabolic stress but also enhances FT fiber recruitment equal to that in high-intensity resistance exercise. According to Henneman’s size principle, slow twitch (ST) fibers with small motor units are predominantly recruited during low-level muscle contraction (Henneman et al. 1965). Despite using low-intensity load, however, BFR exercise could recruit FT fibers to assist the ST fibers because of the maintenance of muscular force during exercise. The additional FT fiber recruitment during BFR exercise may be involved in the decreased oxygen supply and increased metabolic accumulation in working muscle. It is known that resistance training-induced muscular adaptation occurs more extensively in FT fiber than in ST fiber (Charette et al. 1991; McCall et al. 1996), suggesting that FT fiber recruitment during resistance exercise is required to obtain a significant training effect. Therefore, the results of the present study suggest that BFR during low-intensity resistance exercise might be a more effective supplement in multiple set than that in single-set protocols.

As one of its limitations, the present study recruited only healthy young subjects. The majority of previous BFR studies have also examined the training effects on healthy young adult subjects (Clark et al. 2011; Cook et al. 2007; Fujita et al. 2007; Takano et al. 2005, Takarada et al. 2002, 2004). Recent studies (Karabulut et al. 2010, 2011; Ozaki et al. 2011) have examined the effects of BFR exercise in older subjects, and have determined that BFR exercise could effectively lead to muscle hypertrophy and muscle strength increase. Karabulut et al. (2010) reported that the increase of muscle strength induced by BFR exercise was as effective as that of high-intensity resistance training. Moreover, Lanza et al. (2007) reported that the change of intramuscular metabolites during exercise under ischemia was similar in young and older subjects; however, the exercise modality in their study was maximal isometric contraction. Therefore, further study would be needed to determine the metabolic responses during BFR exercise in older subjects or patients with diseases. On the other hand, a previous study (Karabulut et al. 2011) reported that BFR resistance exercise could improve bone marker in blood level equally to high-intensity resistance exercise despite using low-intensity mechanical load. It suggests that BFR resistance exercise is also a potentiality effective training method to bone in addition to muscle. Thus, BFR resistance exercise may be most beneficial for weak individuals such as older people and diseased patients, including chronic heart failure, chronic obstructive pulmonary disease, cancer, and osteoporosis.

In conclusion, the present study showed that multiple-set low-intensity BFR exercise with continuous BFR could achieve intramuscular metabolic stress and skeletal muscle fiber recruitment equal to those of high-intensity resistance exercise.
